# Anatomical Remodeling of the Upper Airway after Laparoscopic Sleeve Gastrectomy: A Multimodal Assessment of Structural and Functional Improvements in Obstructive Sleep Apnea

**DOI:** 10.1007/s11695-025-08352-z

**Published:** 2025-11-13

**Authors:** Mohamed Hany, Mohamed H. Zidan, Mohamed Shawky Elhadidy, Anwar Ashraf Abouelnasr, Mohamed Mahmoud El Shafei, Khaled Matrawy, Ahmed Mostafa Kassem, Asmaa Hamdy, Ehab Elmongui, Toka Aziz El-Ramly, Heba Gharraf, Adel Ibrahim Hozien, Jaidaa Mekky

**Affiliations:** 1 Department of Surgery, Medical Research Institute, Alexandria University, Alexandria, Egypt; 2 Madina Bariatric Center, Madina Womens Hospital, Alexandria, Egypt; 3 Alexandria University, Alexandria, Egypt; 4 The Research Papyrus Lab, Alexandria, Egypt; 5 Department of Diagnostic and Interventional Radiology, Faculty of Medicine, Port Said University, Port Said, Egypt; 6 High Institute of Public Health, Alexandria University, Alexandria, Egypt; 7 Independent Biostatistical Consultant, Alexandria, Egypt; 8 Department of Anesthesia and Surgical Intensive Care, Faculty of Medicine, Alexandria University, Alexandria, Egypt; 9 Department of Diagnostic and Interventional Radiology, Faculty of Medicine,, Alexandria University, Alexandria, Egypt; 10 Department of Chest Diseases, Faculty of Medicine, Alexandria University, Alexandria, Egypt; 11 Department of Neurology, Faculty of Medicine, Alexandria University, Alexandria, Egypt; 12 Department of Anesthesia and Surgical Intensive Care, Medical Research Institute, Alexandria University, Alexandria, Egypt; 13 Department of Diagnostic Radiology, Medical Research Institute, Alexandria University, Alexandria, Egypt

**Keywords:** Metabolic and bariatric surgery, Sleeve gastrectomy, Obstructive sleep apnea, Apnea-hypopnea index, Oxygen desaturation index, Epworth sleepiness scale

## Abstract

**Introduction:**

Obstructive Sleep Apnea (OSA) represents a significant global health challenge, closely linked to obesity and a heightened risk for cardiovascular and metabolic disorders. Continuous Positive Airway Pressure (CPAP) remains the cornerstone of OSA management; however, its effectiveness is often hindered by patient adherence and tolerance. Metabolic and bariatric surgery (MBS) has emerged as a viable alternative by reducing excess weight and improving upper airway anatomy. Among the various MBS techniques, Sleeve Gastrectomy (SG) has gained prominence due to its favorable outcomes and limited complications. This study aims to assess the impact of SG on OSA, utilizing Magnetic Resonance Imaging (MRI) and polysomnography to analyze structural changes and clinical outcomes.

**Methods:**

In this prospective study, 40 participants aged 18–65 years with a BMI exceeding 30 kg/m² and a confirmed diagnosis of OSA (apnea-hypopnea index [AHI] ≥ 5) were enrolled. Pre- and post-operative evaluations included an MRI of the upper airway, polysomnography, and the Epworth Sleepiness Scale (ESS) to quantify daytime somnolence. Statistical analysis was conducted using Generalized Estimating Equations (GEE) and correlation tests in R software, focusing on changes in weight, BMI, AHI, Oxygen Desaturation Index (ODI), and airway dimensions.

**Results:**

Post-SG, participants exhibited significant weight reduction, averaging − 43.8 kg (*p* < 0.001), and a decrease in BMI of -15.7 kg/m² (*p* < 0.001). Both AHI and ODI demonstrated significant declines (*p* < 0.001), with daytime sleepiness normalizing in all subjects. MRI analysis indicated notable increases in upper airway dimensions coupled with a reduction in tongue volume. Additionally, CPAP reliance decreased from 90% to 22.5%. Remarkably, diabetes and hypertension were resolved in all subjects.

**Conclusion:**

This study suggests that SG may yield 12-month improvements in OSA severity, upper airway structure, and CPAP dependency in patients with obesity. The findings highlight SG as a potentially valuable adjunct treatment, although larger, long-term studies are warranted to confirm these results and inform clinical decision-making.

**Supplementary Information:**

The online version contains supplementary material available at 10.1007/s11695-025-08352-z.

## Key Points

SG significantly alleviates OSA and improves daytime sleepiness.

SG led to a 78.9% reduction in the apnea-hypopnea index.

After SG, 77.5% of patients became CPAP-independent.

Postoperative MRI evaluations revealed notable anatomical changes, leading to improved airflow and reduced airway obstruction.

**Supplementary Information:**

The online version contains supplementary material available at 10.1007/s11695-025-08352-z.

## Introduction

In 1837, Charles Dickens introduced the character Joe in “The Pickwick Papers”, one of the earliest literary depictions that aligns with what we now classify as Obstructive Sleep Apnea (OSA). Joe, characterized by being overweight and having daytime somnolence, would later inspire the term “Pickwickian syndrome,” coined by William Osler [1, 2].

In the 1960 s, Gastaut et al. employed early polygraphic techniques to investigate sleep-disordered breathing in obesity, establishing foundational insights into disrupted nocturnal respiration [3]. The advent of polysomnography (PSG) in the 1970 s facilitated Guilleminault et al. in delineating OSA as a specific clinical entity characterized by recurrent upper airway obstructions [4], solidifying OSA’s status as a critical sleep disorder and laying the groundwork for contemporary diagnostic and therapeutic approaches [4].

Currently, OSA is acknowledged as a global health crisis, impacting an estimated 1 billion individuals worldwide [5]. Its rising prevalence coincides with increasing obesity rates posing significant public health challenges, including amplified risks of cardiovascular disease, metabolic disorders, and heightened mortality rates [6–8]. Traditional interventions, such as continuous positive airway pressure (CPAP), are effective yet often necessitate lifelong adherence and can be poorly tolerated by patients [9, 10].

Metabolic and bariatric surgery (MBS) has emerged as a promising treatment option for patients with concurrent obesity and OSA. By directly targeting excessive body weight, MBS has demonstrated significant reduction in OSA severity and, in some cases, complete remission of the condition [11, 12].

Numerous studies have assessed the impact of different MBS procedures on OSA outcomes [13–15]. For instance, Furlan et al. found a remarkable effect after Roux-en-Y gastric bypass (RYGB), with 70.8% of participants achieving remission of OSA [13]. Notably, the prevalence of moderate OSA decreased from 41.7% to 8.3%, and severe OSA dropped from 20.8% to 0% after surgery [13]. Similarly, Zhao et al. reported a significant decline in the apnea-hypopnea index (AHI) among all OSA patients within 3 to 12 months following sleeve gastrectomy (SG) [14]. Further longitudinal analysis by Kikkas et al. indicated a 61.5% remission rate of OSA five years after SG [15], while Currie et al. corroborated these findings with a 56.1% remission rate [16].

Despite the documented clinical improvements attributed to SG, a notable gap in research utilizing imaging modalities, such as Magnetic Resonance Imaging (MRI), to longitudinally assess the structural alterations in the upper airway before and after surgery. Wang et al. employed MRI to reveal that MBS resulted in augmentation of the velopharyngeal airway volume, concomitant with reductions in tongue and pharyngeal lateral wall volumes; however, their study did not establish a direct correlation between these anatomical changes and the functional outcomes gauged through polysomnography [17].

The current study aims to investigate the efficacy of sleeve gastrectomy (SG) in alleviating symptoms of obstructive sleep apnea (OSA) by integrating objective metrics, such as MRI scans of the upper airway and concurrent PSG.

## Patients and Methods

### Study Design and Ethical Considerations

This prospective study was conducted on patients with OSA who were referred to the MBS department at the Medical Research Institute, Alexandria University, Alexandria, Egypt, from May 2023 to January 2024. Ethical approval was granted by the Institutional Review Board (IRB) of the Faculty of Medicine, Alexandria University (Reference Number 0306534). Informed consent was obtained from all participants, and all procedures adhered strictly to established ethical guidelines.

### Eligibility Criteria

Included patients were MBS candidates planned for a primary SG, aged 18 to 65 years, with a BMI of ≥ 30 kg/m², and diagnosed with OSA confirmed via clinical symptomatology and PSG (AHI ≥ 5).

Patients presenting with severe cardiac or pulmonary disorders, significant neurological or psychiatric conditions, severe cognitive impairments, non-obesity-related sleep apnea, patients with obesity hypoventilation syndrome, or other uncontrollable sleep disorders (e.g., parasomnias or narcolepsy) were excluded to maintain the study’s focus on OSA. Additionally, patients with a documented history of gastroesophageal reflux disease, whether previously diagnosed or identified through esophagogastroduodenoscopy (EGD), were also excluded from the study.

### Data Collection and Perioperative Assessment

Demographic details, including sex and age, were systematically collected. BMI was calculated using the formula: BMI = weight/height². Preoperative Clinical evaluation comprised a comprehensive medical history, addressing comorbidities like type 2 diabetes, hypertension, dyslipidemia, osteoarthritis, cardiac disorders, and hypothyroidism, as well as smoking status and physical examination. Preoperative laboratory tests were performed along with an EGD to ascertain anatomical suitability for SG. All smoking participants were mandated to engage in a smoking cessation program before surgery. Laboratory testing included fasting glucose, HbA1c, and lipid profiles (triglycerides, LDL, total cholesterol).

Postoperatively, patients were admitted for a monitoring period of 2 to 4 days to identify potential complications such as anastomotic leaks, hemorrhage, infections, and nutritional deficiencies. Structured follow-up appointments were arranged at 1, 3, 6, and 12 months after surgery for detailed clinical evaluations, weight assessments, laboratory testing, and monitoring of any emergent complications.

### Perioperative Assessment of OSA

The Arabic version of the Epworth Sleepiness Scale (ArESS) [18] was employed to quantify daytime sleepiness and its relationship with sleep-disordered breathing (SDB) in the cohort [18, 19]. For clarity, all ArESS scores are reported as Epworth Sleepiness Scale (ESS) throughout the manuscript, as the questionnaire is structurally equivalent to the standard ESS aside from language adaptation. Relevant laboratory assessments provided insights into associated medical conditions and inflammatory markers [20].

For the physiological evaluation of OSA severity and postoperative changes after SG, a level 3 Polygraphy was leveraged using the MiniScreen PRO (Lowenstien Medical), yielding key metrics for diagnosing OSA and assessing its severity. Specifically, key outcomes such as AHI and Oxygen Desaturation Index (ODI) were recorded, with AHI reflecting the frequency of apneas (complete cessation of airflow) and hypopneas (partial reduction in airflow) per hour of sleep, while ODI quantifies the number of oxygen desaturation events (defined by a decrease in blood oxygen saturation ≥ 3% or 4%) per hour.

Preoperative CPAP therapy was prescribed for all patients with moderate-to-severe OSA (AHI ≥ 15), following the perioperative recommendations from the Society for Ambulatory Anesthesia (SAMBA) 2012 Consensus Statement [21], American Society of Anesthesiologists (ASA) 2006 Practice Guidelines [22], European Respiratory Society (ERS) 2021 Clinical Practice Guidelines [23], and the perioperative enhanced recovery pathways in bariatric surgery consensus [24]. All ESS assessments were performed preoperatively and again at 12 months postoperatively. CPAP was initiated by the referring pulmonologist and typically maintained for at least three months before surgical referral. Concurrent risk optimization, including smoking cessation and metabolic control, was undertaken to ensure safe surgical candidacy. CPAP therapy was assessed to determine its effectiveness in managing OSA by monitoring changes in CPAP requirements following surgery and establishing the discontinuation of CPAP use upon observing significant clinical improvements.

The severity of daytime sleepiness was classified into four categories [25]: Normal: < 10; Mild: 11–12; Moderate: 13–15; and Severe: ≥ 16. Concurrently, OSA severity was classified based on the AHI and the ODI using the same categorical framework [26]: Normal: < 5 events per hour; Mild: 5–14 events; Moderate: 15–29 events; and Severe: ≥ 30 events per hour. CPAP discontinuation was guided by postoperative clinical improvement, AHI values, physician evaluation, and patient-reported symptom remission. Patients who achieved an AHI < 15 events/hour and no longer exhibited clinical symptoms of OSA were considered eligible for discontinuation of CPAP, consistent with current ERS guidelines [23].

All patients underwent overnight PSG testing at baseline (pre-operatively) and again 12 months post-operatively. Electrodes were placed according to the manufacturer’s standard guidelines. The studies were conducted in the sleep laboratory of the Department of Neurology using the Cadwell data acquisition system (Version 2.1, USA).

### MRI Assessment of the Upper Airway

Magnetic Resonance Imaging (MRI) was utilized to analyze upper airway morphology both pre- and postoperatively, employing a 1.5T TOSHIBA Titan scanner, Japan. The imaging protocol encompassed a series of scans from the posterior nasal spine to the hyoid bone, with images interpreted by a qualified radiologist. The specific sequences included sagittal T2 TSE, STIR, and T1 TSE; coronal T1 TSE (each with a slice thickness of 3.5 mm and no interslice gap); and axial T2 SPIR, T1 TSE, and DWI sequences (with a slice thickness of 3 mm and a 1.5 mm interslice gap).

Key anatomical parameters evaluated in the airway included Maximum Length of the Soft Palate (MLSP), Maximum Thickness of the Soft Palate (MTSP), Posterior Nasal Airway Space (PNAS), Occlusal Posterior Airway Space (OPAS), Mandibular Posterior Airway Space (MPAS), and tongue volumes. Measurements were conducted using the RadiAnt DICOM Viewer [27].

Tongue morphology was quantitatively assessed through the measurement of the sagittal diameter, which is defined as the maximal anteroposterior dimension from the apex to the base of the tongue, alongside thickness, measured as the longest distance from the origin of the genioglossus muscle to the tongue surface [28]. Additionally, coronal and axial dimensions were obtained as the greatest laterolateral measurements on coronal T1 TSE and axial T2 SPIR and T1 TSE images. All analyses were grounded on T2- and T1-weighted sequences to maintain uniformity in anatomical delineation [28].

### Surgical Technique

Laparoscopic Sleeve gastrectomy (SG) was selected as the surgical intervention due to its widespread use, relative technical simplicity, and lower risk of nutritional deficiencies and surgical complications compared to malabsorptive procedures such as Roux-en-Y gastric bypass (RYGB) and One-Anastomosis Gastric Bypass (OAGB). Restricting the study to SG ensured procedural uniformity, which was essential for isolating the effects of a single surgical modality on upper airway remodeling.

Standard 5 ports were used: three 12-mm ports (for the camera, right and left working ports) and two 5-mm ports (for liver retraction and the assistant). Pneumoperitoneum was created after using optical trocars for entry. The greater omentum was dissected off the greater curvature of the stomach using the EnSeal device (Ethicon Endo-Surgery, Cincinnati, OH, USA), followed by dissection of any posterior gastric adhesions and excision of Belsey’s pad of fat. The gastric sleeve was created over a 36-Fr calibration bougie using an Echelon Flex Endopath 60-mm linear stapler (Ethicon Endo-Surgery, Cincinnati, OH, USA) for gastric division, starting at 3–5 cm before the pylorus, up to the angle of His using green, gold, and blue reloads according to the thickness of tissues. The staple line was invaginated completely by running seromuscular stitches using unidirectional absorbable 3/0 V-Loc 180 sutures (Covidien, Mansfield, MA, USA). In the case of an incidental intraoperative hiatal hernia, a cruroplasty was done before gastric stapling.

All procedures were conducted under general anesthesia, accompanied by standard perioperative monitoring protocols. There were no complications linked to either the anesthesia or the surgical intervention. Minor intraoperative incidents, including transient hypotension that necessitated vasopressor administration and brief episodes of oxygen desaturation that were corrected through ventilatory adjustments, were addressed promptly and did not disrupt the surgical workflow or postoperative recovery. The postoperative course was uneventful, and minor complications such as superficial wound infections were managed conservatively, resulting in no significant impact on recovery.

### Sample Size Calculation

The sample size was calculated using the ‘pwr.t.test’ function from the ‘pwr’ package in R, specifying a paired t-test with a medium effect size (Cohen’s d = 0.5), two-sided significance level of 0.05, and power of 80%. The calculation indicated that a minimum of 34 paired observations would be required (*n* = 33.37). To account for potential attrition and maintain adequate statistical power for detecting pre- and post-treatment differences, a total of 40 patients were enrolled.

### Statistical Analysis

Statistical analyses were performed using R software version 4.4.2. Demographic and clinical characteristics were summarized using descriptive statistics, including means, standard errors, medians, ranges, frequencies, and percentages. To evaluate changes in continuous outcomes such as weight, BMI, AHI, ODI, ESS, and upper airway MRI parameters from pre- to post-surgery, we used Generalized Estimating Equations (GEE).

To assess whether the magnitude of improvement following sleeve gastrectomy (SG) differed according to obesity class or preoperative CPAP duration, generalized estimating equation (GEE) models were fitted with an exchangeable correlation structure and identity link. Each model included a main effect for time (pre- vs. post-surgery), the grouping variable (obesity class or CPAP duration), and their interaction.

For the obesity-based comparison, Obesity class was defined according to WHO guidelines: Class I (BMI 30.0–34.9 kg/m²), Class II (35.0–39.9 kg/m²), and Class III (≥ 40 kg/m²). For subgroup comparisons, Class I and II were combined and contrasted with Class III. The interaction term (Time × Obesity Class) estimated the difference-in-differences (DiD) in outcome changes between patients with Class III obesity and those with combined Class I–II. For the CPAP analysis, the interaction term (Time × CPAP Duration) estimated the additional change in each outcome per one-month increase in preoperative CPAP use. CPAP duration was modeled as a continuous variable, with non-users assigned a value of zero. All models were applied separately to each outcome. Results are presented as adjusted mean differences (MDs) with corresponding 95% confidence intervals and p-values, with significance defined as *p* < 0.05.

Pearson correlation coefficients were calculated to examine the association between percentage total weight loss (%TWL) and changes in AHI, ODI, and ESS scores. Influential observations were assessed using Cook’s Distance from linear regression models relating %TWL to changes in AHI, ODI, and ESS. Although none of the observations exceeded the conventional threshold of 1, and all Cook’s Distance percentiles under the F-distribution were below 20%, the top three observations with the highest Cook’s Distance values in each model also exceeded the rule-of-thumb threshold of 4/(N-k-1), where n is the sample size and k is the number of predictors. Notably, these were the same three observations across all models. While they were not considered highly influential, a sensitivity analysis excluding them was conducted to assess the robustness of the results.

For binary categorical outcomes (e.g., CPAP requirement, comorbidity remission), McNemar’s test was applied to assess within-subject changes. While some clinical endpoints—such as remission of hypertension (*n* = 9)—involved small subsample sizes, p-values were calculated using exact methods appropriate for paired binary data. For multi-category variables, including OSA severity (based on AHI and ODI) and daytime sleepiness (ESS categories), Cochran’s Q test was employed. All statistical tests were two-tailed, and significance was defined as *p* < 0.05.

## Results

### Baseline Demographics

A cohort of 40 patients diagnosed with OSA who underwent SG was included in this study, with a notable female predominance (75%). The mean body mass index (BMI) of the participants was 44.9 ± 1.3 kg/m² (Table 1). The median time elapsed since operation was 14 months, with a range of 12 to 15 months. The prevalence of common medical-associated problems among the cohort was 52.5% for osteoarthritis, 35% for dyslipidemia, and 22.5% for hypertension (Table 1).

**Table 1:** Baseline characteristics of the study sample (*N* = 40)

Characteristics	Value (*n*(%) or M ± SE (Range))
**Age**	36.0 ± 1.7 (19.0–59.0)
**Sex**	
Female	30 (75)
Male	10 (25)
**Anthropometrics**	
Weight (Kg)	125.6 ± 4.4 (87.1–217.7)
Height (cm)	166.9 ± 1.5 (153.0–202.0)
BMI (kg/m^2^)	44.9 ± 1.3 (34.8–66.7)
Class I (BMI 30–34.9.9)	1 (2.5)
Class II (BMI 35–39.9.9)	13 (32.5)
Class III (BMI ≥ 40)	26 (65)
**Smoking**	8 (20)
**Associated medical conditions**	
Osteoarthritis	21 (52.5)
Dyslipidemia	14 (35)
Diabetes	7 (17.5)
Hypothyroidism	7 (17.5)
Hypertension	9 (22.5)
Cardiac	3 (7.5)
**CPAP used before SG**	
CPAP need	36 (90)
CPAP duration before SG (months)	6.6 ± 2.9 (3.0–12.0)
CPAP settings (cm H_2_O)	12.3 ± 0.2 (10.0–15.0)

*SG * Sleeve Gastrectomy, *BMI * Body Mass Index, *CPAP * Continuous Positive Airway Pressure. Cell values represent frequency and percentages n(%) or: mean ± standard error (M ± SE) and Range

### Pre-operative OSA Assessment

According to the Epworth Sleepiness Scale (ESS), the majority of participants (75%) exhibited severe daytime sleepiness, with no individuals scoring within the normal range (Table 2). PSG results revealed a predominance of severe sleep-disordered breathing, with 65% of patients having a severe AHI of ≥ 30, while 25% exhibited moderate AHI and 10% presented mild AHI; no patients had a normal AHI (Table 2). Nocturnal oximetry indicated severe desaturation (ODI ≥ 30) in 55% of subjects, while moderate and mild desaturation were identified in 30% and 15%, respectively (Table 2). Preoperative CPAP therapy was necessary for 90% of patients, with an average titrated pressure of 12.3 cmH₂O (Table 2).

**Table 2:** Impact of sleeve gastrectomy (SG) on upper airway function, sleep-disordered breathing (SDB), and medically associated problems in patients with pre- and post-SG

Variable	Pre-operative M ± SE (Range)	Post-operative M ± SE (Range)	MD (95% CI) (After-Before)	*p*
Anthropometrics				
Weight (Kg)	125.6 ± 4.4 (87.1–217.7)	81.8 ± 2.5 (59.0–138.5)	−43.8 (−53.6, −34.0)	< 0.001*
BMI (Kg/m^2^)	44.9 ± 1.3 (34.8–66.7)	29.3 ± 0.7 (22.1–40.5)	−15.7 (−18.5, −12.8)	< 0.001*
% TWL (95% CI)	34.0% (31.5%, 36.6%)			
**MRI of the upper airway**				
Maximum length of the soft palate (mm)	40.8 ± 0.4 (38.0–45.0)	35.9 ± 0.4 (32.0–43.0)	−4.9 (−5.9, −3.8)	< 0.001*
Maximum thickness of soft palate (mm)	12.5 ± 0.2 (10.0–15.0)	9.5 ± 0.2 (8.0–12.0)	−3.0 (−3.6, −2.4)	< 0.001*
Nasal posterior airway space (mm)	5.9 ± 0.2 (4.0–8.0)	8.8 ± 0.2 (7.0–12.0)	3.0 (2.4, 3.5)	< 0.001*
Occlusal posterior airway space (mm)	4.1 ± 0.2 (2.0–6.0)	7.5 ± 0.2 (5.0–10.0)	3.5 (2.9, 4.0)	< 0.001*
Mandibular posterior airway space (mm)	7.8 ± 0.2 (6.0–10.0)	11.7 ± 0.2 (10.0–14.0)	3.9 (3.4, 4.3)	< 0.001*
Tongue volume (mL)	102.5 ± 1.9 (82.0–120.0)	79.2 ± 1.4 (65.0–100.0)	−23.4 (−27.9, −18.9)	< 0.001*
**Daytime Sleepiness**				
Epworth Sleepiness Scale	17.0 ± 0.5 (11.0–22.0)	5.4 ± 0.2 (2.0–8.0)	−11.6 (−12.6, −10.6)	< 0.001*
Normal (< 10)	0 (0)	40 (100)		< 0.001*
Mild (11–12)	4 (10)	0 (0)		
Moderate (13–15)	6 (15)	0 (0)		
Severe (≥ 16)	30 (75)	0 (0)		
**Polysomnography**				
Apnea-Hypopnea Index (events per hour)	38.1 ± 2.5 (10.0–62.0)	8.0 ± 0.6 (2.0–16.0)	−30.1 (−35.0, −25.1)	< 0.001*
Normal (< 5)	0 (0)	7 (17.5)		< 0.001*
Mild (5–14)	4 (10)	30 (75)		
Moderate (15–29)	10 (25)	3 (7.5)		
Severe (≥ 30)	26 (65)	0 (0)		
**Nocturnal oximetry**				
Oxygen Desaturation Index (events per hour)	30.7 ± 2.2 (6.0–51.0)	5.2 ± 0.5 (0.0–11.0)	−25.6 (−30.0, −21.1)	< 0.001*
Normal (< 5)	0 (0)	21 (52.5)		< 0.001*
Mild (5–14)	6 (15)	19 (47.5)		
Moderate (15–29)	12 (30)	0 (0)		
Severe (≥ 30)	22 (55)	0 (0)		
**Continuous Positive Airway Pressure (CPAP) Compliance**				
CPAP need	32 (80)	9 (22.5)		< 0.001*
CPAP settings	12.3 ± 0.2 (10.0–15.0)	7.0 ± 0.4 (5.0–9.0)	−5.3 (−6.2, −4.5)	< 0.001*

*SG* Sleeve Gastrectomy, *BMI * Body Mass Index, *%TWL* Percent Total Weight Loss, *SDB * Sleep-Disordered Breathing, *AHI * Apnea–Hypopnea Index, *ODI * Oxygen Desaturation Index, *CPAP * Continuous Positive Airway Pressure. Data are presented as mean ± standard error (SE) with range, unless otherwise indicated. MD = Mean Difference in outcome between post- and pre-operative measurements, with 95% Confidence Interval (CI). p-values are based on paired comparisons using generalized estimating equations between pre- and post-SG values. Categorical sleep severity classifications are based on standard thresholds for Epworth Sleepiness Scale, AHI, and ODI. *Statistically significant (*p* < 0.05)

### Post-operative OSA Assessment

**Fig. 1:**
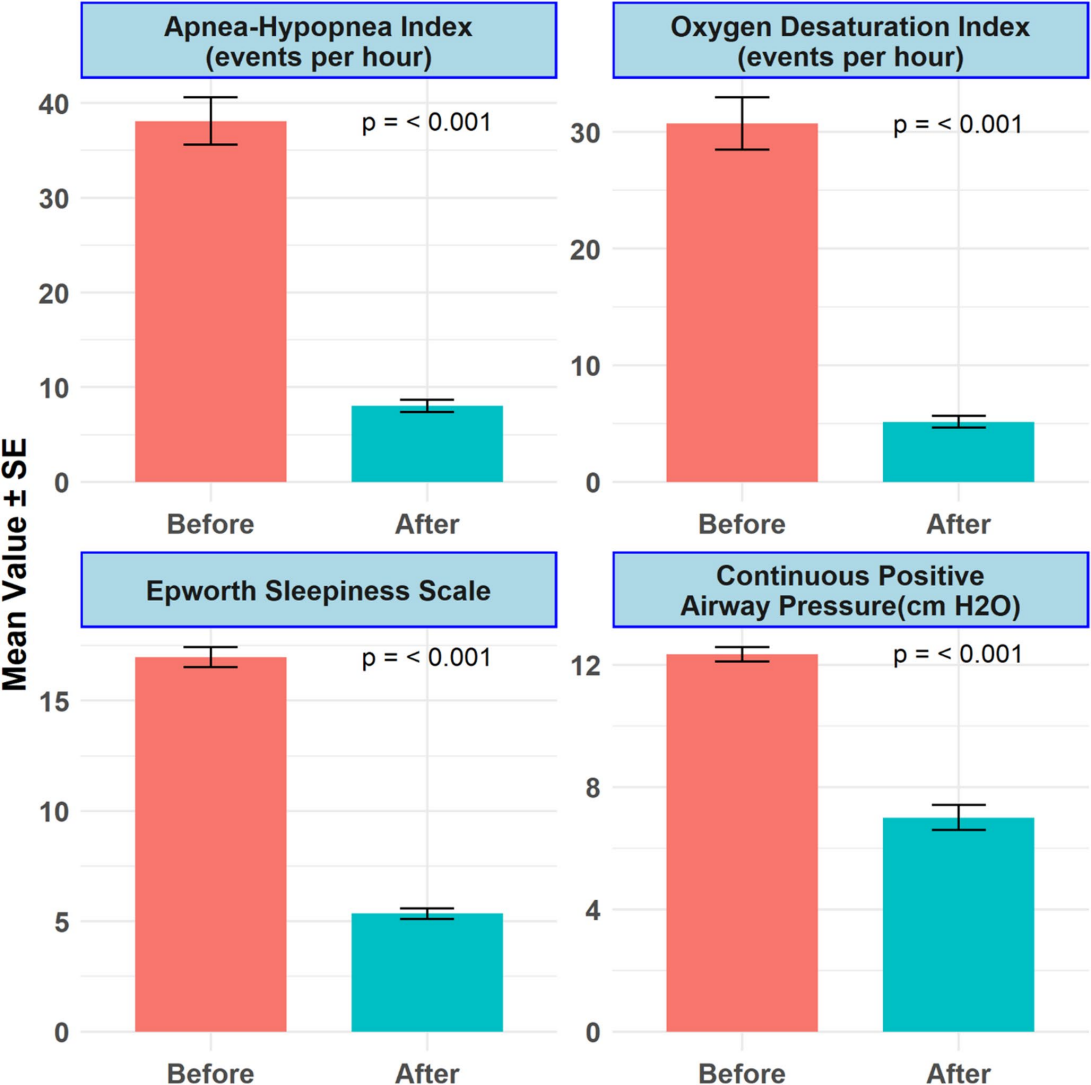
Improvement in sleep-disordered breathing parameters and CPAP requirements following MBS. The graphs depict the mean values ± standard error (SE) for the Apnea-Hypopnea Index (AHI), Oxygen Desaturation Index (ODI), Epworth Sleepiness Scale (ESS), and required Continuous Positive Airway Pressure (CPAP) settings. Significant decreases were observed postoperatively in the AHI, ODI, and ESS, indicating reduced sleep-disordered breathing and daytime sleepiness (all *p* ≤ 0.001). Additionally, there was a substantial reduction in the mean CPAP settings required by patients, reflecting improved respiratory function during sleep (*p* ≤ 0.001)

**Fig. 2:**
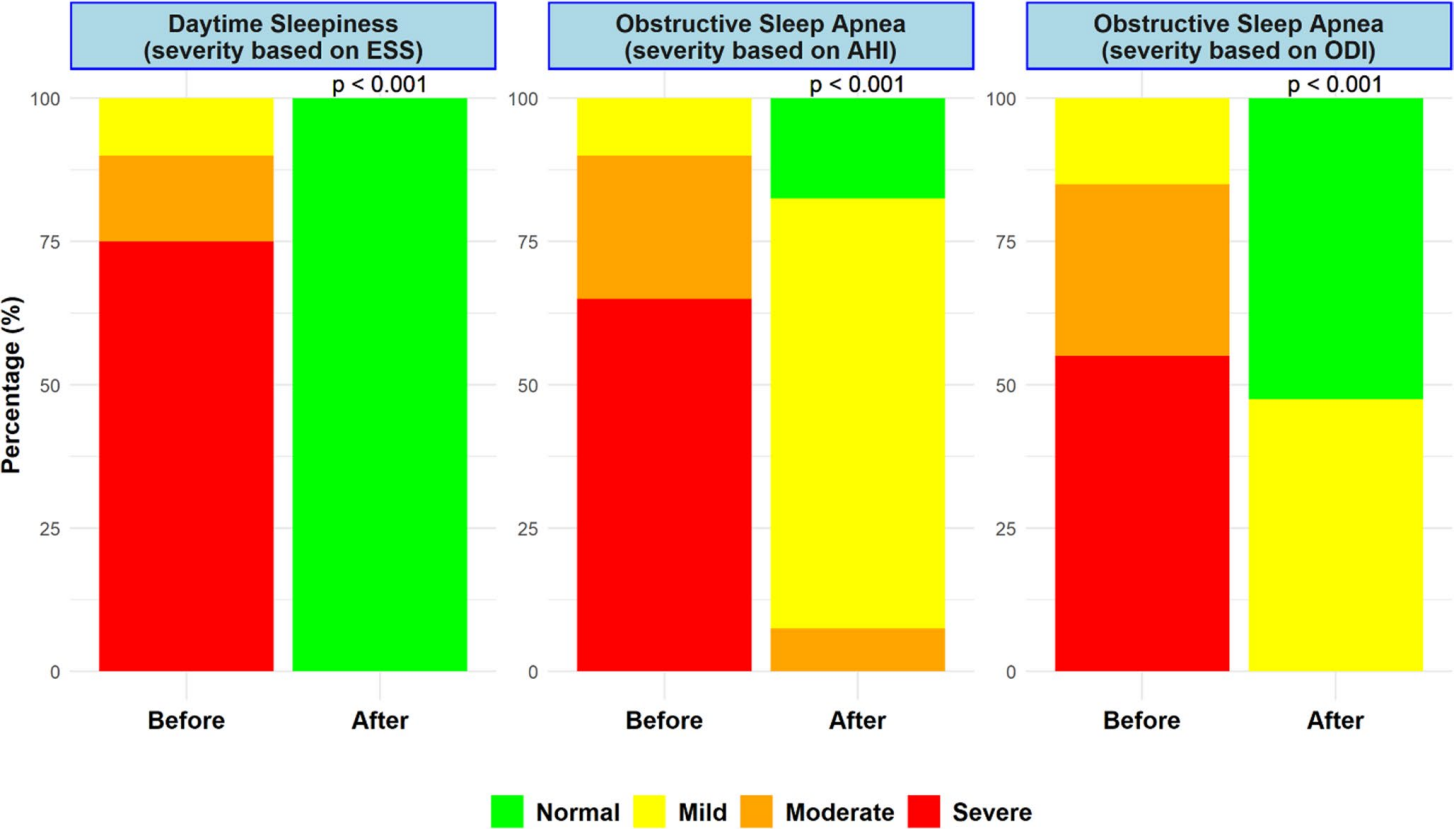
Comparative Analysis of Severity Distribution Before and After SG. This figure illustrates the significant impact of SG on the severity of daytime sleepiness (as measured by ESS) and OSA (as assessed by AHI and ODI). The stacked bar charts indicate a significant shift from higher severity levels (Moderate and Severe) to Normal and Mild across all measures post-surgery, as indicated by a p-value of less than 0.001. SG – Sleeve Gastrectomy; ESS – Epworth Sleepiness Scale; AHI – Apnea-Hypopnea Index; ODI – Oxygen Desaturation Index; OSA – Obstructive Sleep Apnea

Post-operatively at 12-months, patients demonstrated a significant reduction in the ESS from 17.0 ± 0.5 to 5.4 ± 0.2, with a mean difference of −11.6 (CI: −12.6, −10.6, *p* < 0.001) (Fig. 1). Remarkably, all cases of severe OSA were resolved, and the distribution shifted markedly: 75% (*n* = 30) of patients were classified as having mild OSA, 7.5% (*n* = 3) had moderate OSA, and 17.5% (*n* = 7) achieved complete remission, defined as an AHI < 5. PSG illustrated a substantial decrease in AHI from 38.1 ± 2.5 events per hour to 8.0 ± 0.6 (MD: −30.1, CI: −35.0, −25.1, *p* < 0.001) (Fig. 2) (Table 2).

Similarly, the oxygen desaturation index (ODI) improved substantially. Preoperatively, 55% of patients had severe ODI (≥ 30), 30% had moderate ODI (15–29), and 15% had mild ODI (5–14). Postoperatively, 52.5% (*n* = 21) of patients achieved normalization (ODI < 5), while 47.5% (*n* = 19) remained in the mild category. No patients had moderate or severe ODI postoperatively (*p* < 0.001) (Fig. 2) (Table 2). This categorical shift underscores a substantial improvement in OSA severity, with statistically significant reductions across all severity levels.

There was a significant reduction in the necessity for CPAP, dropping from 90% to 22.5% (*p* < 0.001), with a concurrent reduction in mean titrated pressure from 12.3 ± 0.2 to 7.0 ± 0.4 cm H₂O (Table 2) (Fig. 1).

Metabolic parameters also demonstrated notable enhancements, with average reductions in fasting glucose (15.4 mg/dL) and HbA1c (0.6%). Lipid profiles showed average decreases in triglycerides (−44.0 mg/dL), LDL cholesterol (−17.4 mg/dL), and total cholesterol (−26.8 mg/dL) (Table 2).

Moreover, a remarkable remission of comorbid conditions was observed postoperatively (Table 3). All cases of diabetes and hypertension were resolved (*p* = 0.023 and *p* = 0.008, respectively). Improvements in hypothyroidism and dyslipidemia were observed in 85.7% of cases, although this did not reach statistical significance (*p* = 0.077 and *p* = 0.480, respectively). Osteoarthritis showed improvements in all patients (100%), while cardiac conditions resolved in 66.7% of cases, with improvements noted in 33.3%, though neither outcome achieved statistical significance (*p* = 0.480 for both) (Table 3).

**Table 3:** Impact of MBS on the associated medical conditions

Condition	Before	After	*P*-value
Osteoarthritis	21 (52.5)	21 (52.5)	
Dyslipidemia	14 (35)	12 (30)	0.480
Diabetes	7 (17.5)	0 (0)	0.023*
Hypothyroidism	7 (17.5)	1 (2.5)	0.077
Hypertension	9 (22.5)	0 (0)	0.008*
Cardiac	3 (7.5)	1 (2.5)	0.480

Cell values represent frequency and percentages n(%)

*statistically significant differences at *p* < 0.05

### MRI Analysis and Postoperative Structural Changes

**Fig. 3:**
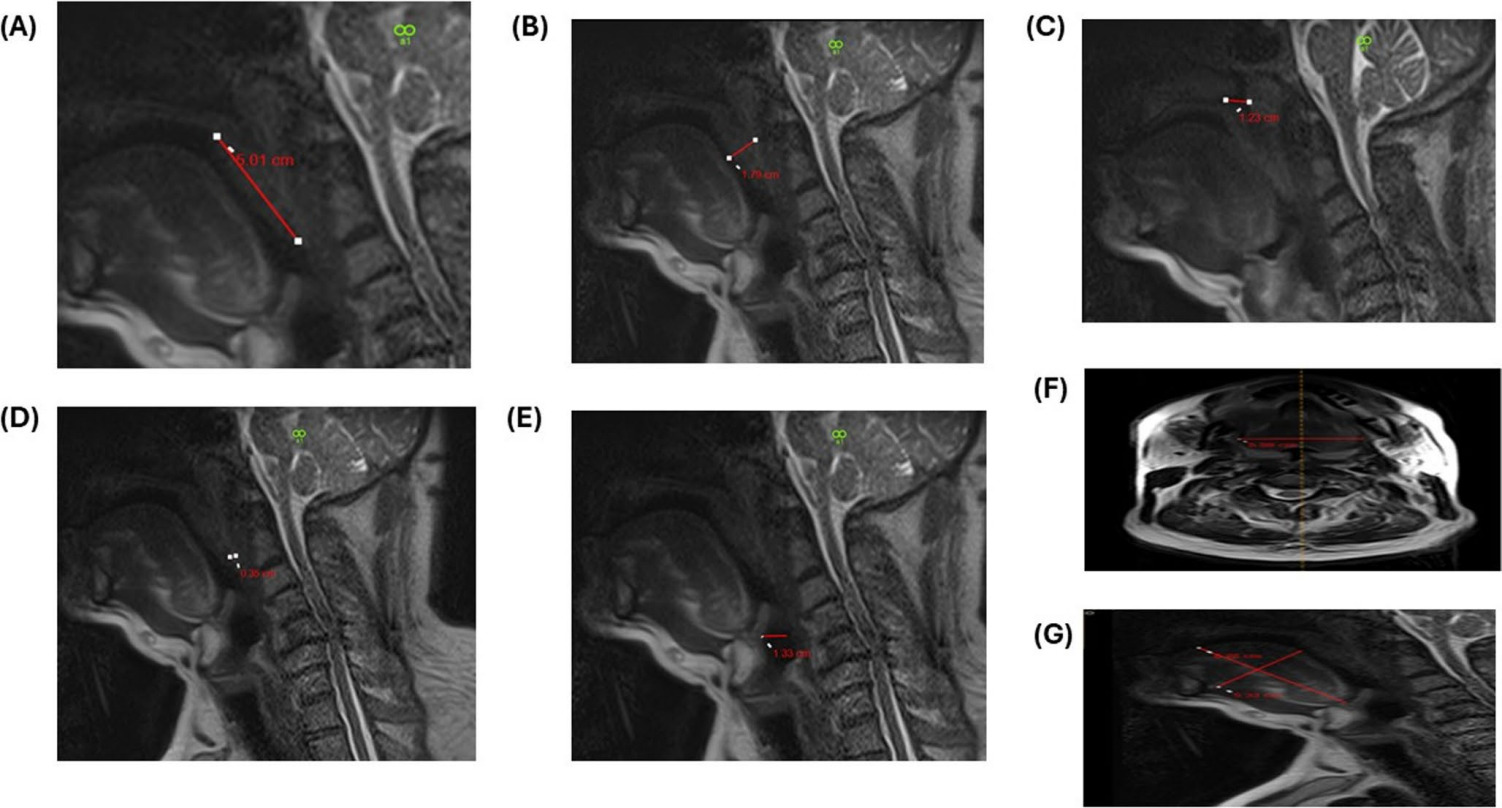
Pre-operative MRI images for assessment of the same patient showing (**A**) a maximum length of the soft palate (MLSP) of 50.1 mm, (**B**) a maximum thickness of the Soft palate (MTSP) of 17.9 mm, (**C**) Posterior nasal airway space (PNAS) of 12.3 mm, (**D**) Occlusal posterior airway space (OPAS) of 3.5 mm, (**E**) mandibular posterior airway space (MPAS) of 13.3 mm, and (**F**) coronal tongue dimensions of 5.59 mm and (**G**) axial tongue dimensions of 8.76 × 5.33 mm for an estimated tongue volume of 137mL. MRI – Magnetic Resonance Imaging

Pre-operative MRI evaluations delineated significant anatomical constraints contributing to upper airway collapse and obstruction in patients with OSA (Fig. 3). PNAS measurements averaged 5.9 ± 0.2 mm, markedly below the normative threshold of 11 mm. Tongue volume was substantially enlarged, with a mean of 102.6 ± 1.9 mL, exceeding the expected range of 70–90 mL. Furthermore, the dimensions of the soft palate demonstrated pronounced hypertrophy, with the MLSP averaging 40.8 ± 0.4 mm and the MTSP measuring 12.5 ± 0.2 mm. Both OPAS and MPAS demonstrated significant constriction, measuring 4.1 ± 0.2 mm and 7.9 ± 0.2 mm, respectively (Table 1).

**Fig. 4:**
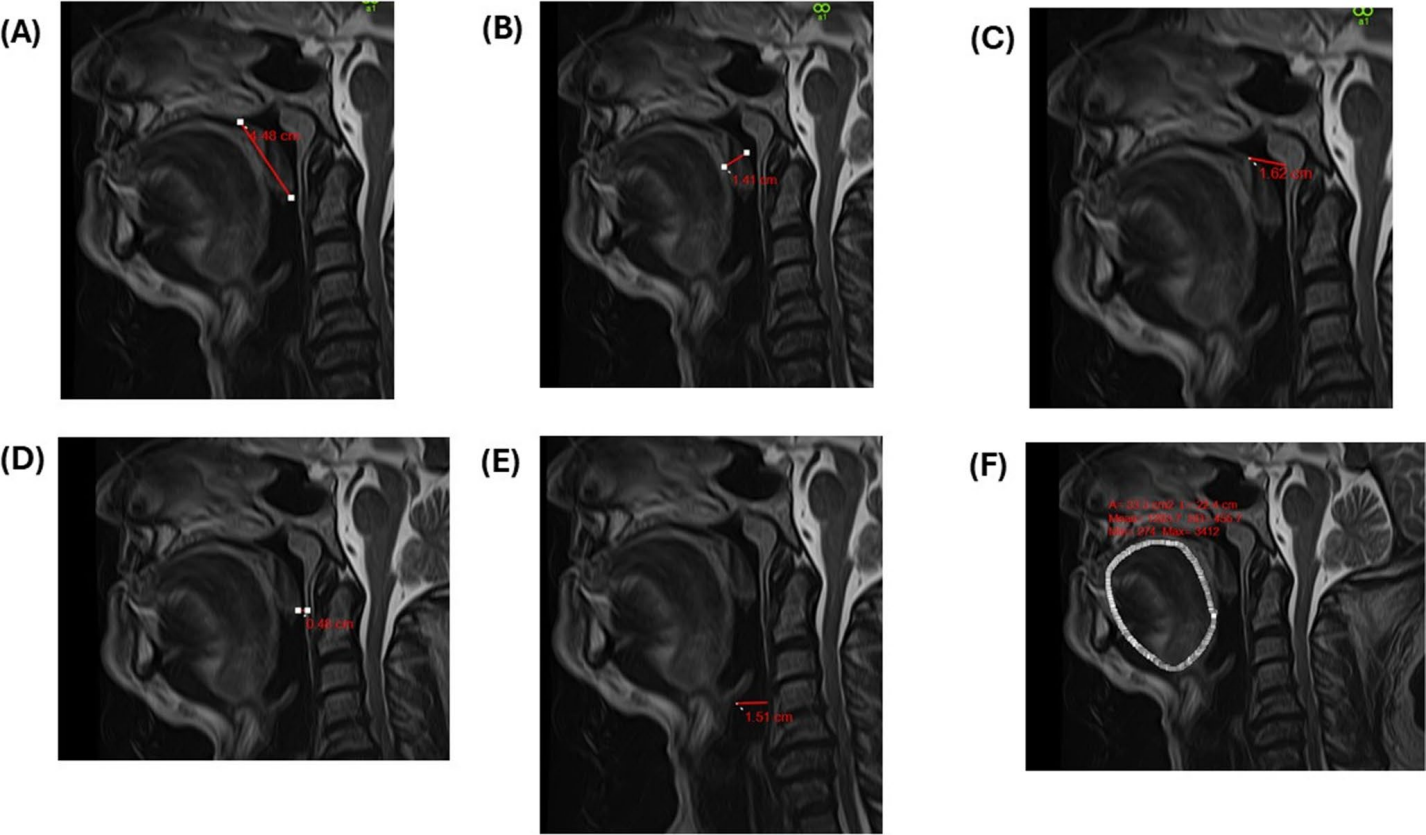
Postoperative MRI images for assessment of the same patient illustrated in Fig. 3 with a postoperative (**A**) MLSP of 44.8 mm, (**B**) MTSP of 14.1 mm, (**C**) PNAS of 16.2 mm, (**D**) OPAS of 4.8 mm, (**E**) MPAS of 15.1 mm, and (**F**) tongue Volumes with a maximum volume of 74.6mL. MRI – Magnetic Resonance Imaging; MLSP – Maximum Length of the Soft Palate; MTSP – Maximum Thickness of the Soft Palate; PNAS – Posterior Nasal Airway Space; OPAS – Occlusal Posterior Airway Space; MPAS – Mandibular Posterior Airway Space

**Fig. 5:**
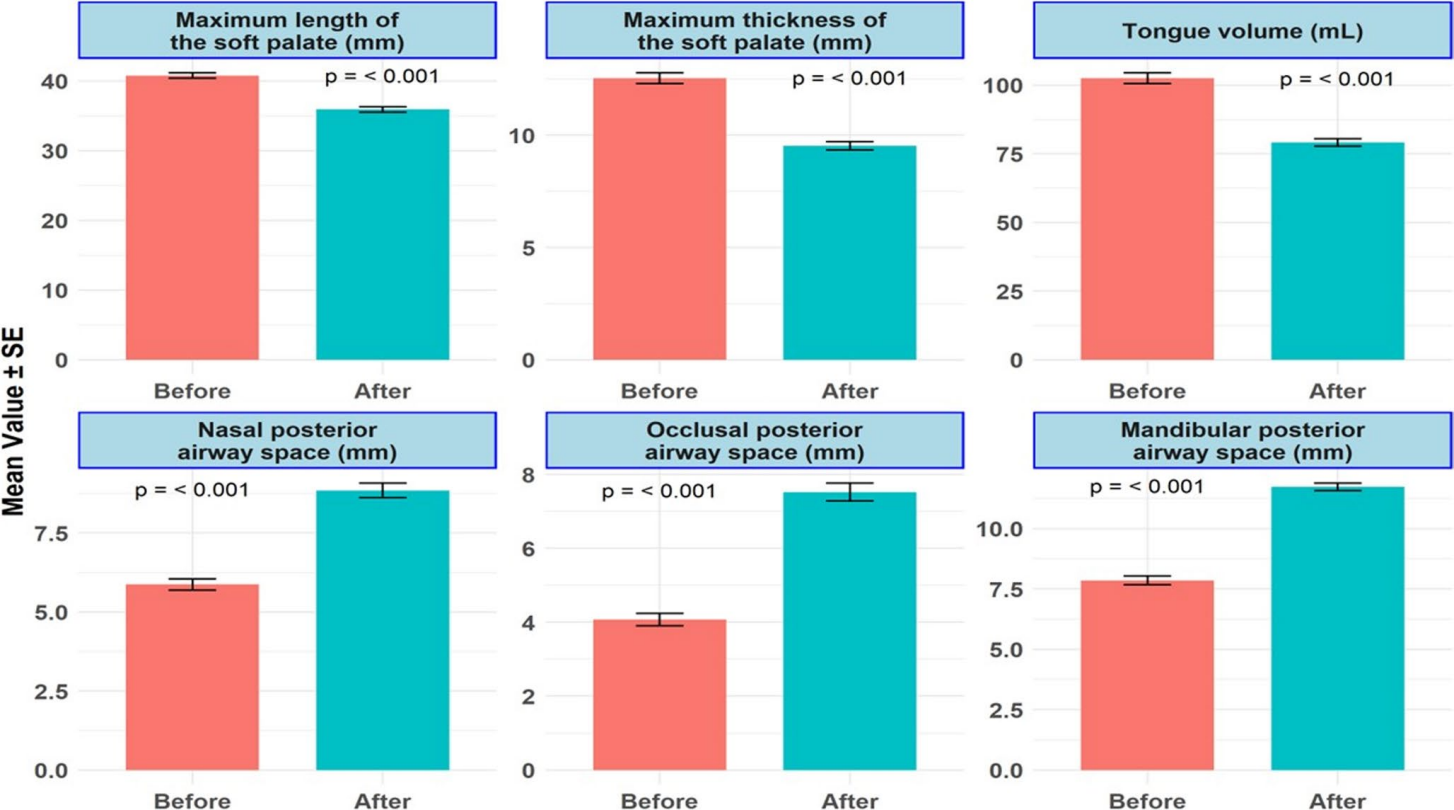
Changes in upper airway dimensions and tongue volume before and after MBS. The graphs display mean values ± standard error (SE) for the maximum length and thickness of the soft palate, nasal posterior airway space, occlusal posterior airway space, mandibular posterior airway space, and tongue volume. Statistically significant reductions were observed in the maximum length and thickness of the soft palate and tongue volume, while significant increases were noted in the nasal, occlusal, and mandibular posterior airway spaces (all *p* ≤ 0.001). MBS – Metabolic and Bariatric Surgery

Post-operative MRI analyses, conducted 12 months after SG, revealed a profound anatomical remodeling associated with improved upper airway patency (Fig. 4). PNAS demonstrated a significant increase to 11.3 ± 0.4 mm (*p* < 0.001) (Fig. 5). Tongue volume exhibited a marked reduction of 18.5%, decreasing from 102.6 ± 1.9 mL to 83.7 ± 1.5 mL (*p* < 0.001), indicative of a substantial reduction in soft tissue bulk at the tongue base, thereby mitigating posterior airway collapse. Notably, MLSP and MTSP dimensions demonstrated significant reductions postoperatively, with MLSP decreasing to 36.2 ± 0.3 mm and MTSP to 10.8 ± 0.2 mm (*p* < 0.001), highlighting the alleviation of soft tissue crowding in the oropharyngeal region (Fig. 5). Concomitant improvements in OPAS and MPAS were observed, with OPAS expanding to 7.4 ± 0.3 mm and MPAS increasing to 11.2 ± 0.4 mm (*p* < 0.001), reflecting enhanced posterior airway patency and improved airflow dynamics (Table 2) (Fig. 5).

### Subgroup Comparison of Obesity Classes on Postoperative Improvements in Airway Anatomy and Sleep-Disordered Breathing after SG

To explore whether postoperative improvements varied by baseline obesity severity, we stratified patients into Class III (BMI ≥ 40 kg/m²) and Class I–II (BMI 30–39.9 kg/m²) categories and conducted generalized estimating equation (GEE) analyses. No statistically significant differences were observed in the magnitude of change for any MRI-based upper airway measurements, including soft palate length (DiD: 0.19 mm, 95% CI: − 1.86 to 2.24; *p* = 0.854), soft palate thickness (DiD: − 0.44 mm, 95% CI: − 1.67 to 0.79; *p* = 0.484), and nasal posterior airway space (DiD: − 0.15 mm, 95% CI: − 1.30 to 1.00; *p* = 0.801) (Table 4). Similarly, functional outcomes such as the ESS, AHI, ODI, and CPAP pressure requirements exhibited comparable improvements across obesity classes, indicating that SG-mediated anatomical and functional benefits were not modified by baseline BMI category.

**Table 4:** Difference-in-Differences estimates comparing the impact of class III versus class I–II obesity on changes in MRI-Based airway Measures, Sleep-Disordered breathing Indices, and CPAP settings after sleeve gastrectomy

Variable	DiD (95% CI) (Class III – Class I/II)	*p*
MRI of upper airway		
Maximum length of the soft palate (mm)	0.19 (−1.86, 2.24)	0.854
Maximum thickness of soft palate (mm)	−0.44 (−1.67, 0.79)	0.484
Nasal posterior airway space (mm)	−0.15 (−1.30, 1.00)	0.801
Occlusal posterior airway space (mm)	−0.19 (−1.32, 0.94)	0.746
Mandibular posterior airway space (mm)	0.47 (−0.45, 1.38)	0.318
Tongue volume (mL)	1.36 (−7.75, 10.47)	0.769
**Daytime Sleepiness**		
Epworth Sleepiness Scale	−0.08 (−2.03, 1.87)	0.934
**Polysomnography**		
Apnea-Hypopnea Index (events per hour)	−1.62 (−11.50, 8.27)	0.749
**Nocturnal oximetry**		
Oxygen Desaturation Index (events per hour)	−1.18 (−10.14, 7.79)	0.797
**CPAP settings**	−0.58 (−1.97, 0.81)	0.410

*DiD* Difference-in-Differences, *CI * Confidence Interval, *SG * Sleeve Gastrectomy, *CPAP * Continuous Positive Airway Pressure, *AHI * Apnea–Hypopnea Index, *ODI * Oxygen Desaturation Index. Estimates reflect interaction terms (Time × Obesity Class) from generalized estimating equation (GEE) models using an exchangeable correlation structure. Each DiD value represents the additional change in outcome from pre- to post-surgery in Class III obesity relative to Class I–II. Negative values indicate reduced improvement in Class III; positive values indicate greater improvement in Class III except for upper airway spaces. Results should be interpreted cautiously due to the small subgroup sample sizes (Class I–II: *n* = 14; Class III: *n* = 26)

### Effect of Preoperative CPAP Duration on Postoperative Improvements in Airway Anatomy and Sleep-Disordered Breathing

We further assessed whether the magnitude of improvement was influenced by the duration of preoperative CPAP therapy. In this analysis, CPAP duration was treated as a continuous variable (in months), with non-users assigned a value of zero. Longer preoperative CPAP use was significantly associated with reduced improvements in several outcomes (Table 5). Specifically, each additional month of CPAP use was associated with smaller reductions in ESS (MD = − 0.26 points/month, 95% CI: − 0.45 to − 0.06; *p* = 0.010), AHI (MD = − 2.39 events/hour/month, 95% CI: − 3.17 to − 1.61; *p* < 0.001), and ODI (MD = − 2.08 events/hour/month, 95% CI: − 2.77 to − 1.39; *p* < 0.001) (Table 5). Anatomical improvements showed similar trends: longer CPAP use predicted a significantly smaller decrease in soft palate length (MD = − 0.30 mm/month, 95% CI: − 0.55 to − 0.04; *p* = 0.026), while other MRI-based parameters demonstrated non-significant trends in the same direction (Table 5).

**Table 5:** Adjusted mean differences per One-Month increase in preoperative CPAP use on changes in MRI-Based airway Measures, Sleep-Disordered breathing Indices, and CPAP settings after versus before sleeve gastrectomy

Variable	Adjusted MD (95% CI) per 1-Month Increase in Preoperative CPAP Use	*p*
MRI of upper airway		
Maximum length of the soft palate (mm)	−0.30 (−0.55, −0.04)	***0.026***
Maximum thickness of soft palate (mm)	−0.08 (−0.19, 0.03)	0.160
Nasal posterior airway space (mm)	0.00 (−0.12, 0.13)	0.945
Occlusal posterior airway space (mm)	−0.06 (−0.17, 0.06)	0.323
Mandibular posterior airway space (mm)	0.04 (−0.08, 0.17)	0.478
Tongue volume (mL)	−0.85 (−1.83, 0.14)	0.092
**Daytime Sleepiness**		
Epworth Sleepiness Scale	−0.26 (−0.45, −0.06)	***0.010***
**Polysomnography**		
Apnea-Hypopnea Index (events per hour)	−2.39 (−3.17, −1.61)	***< 0.001***
**Nocturnal oximetry**		
Oxygen Desaturation Index (events per hour)	−2.08 (−2.77, −1.39)	***< 0.001***
**CPAP settings**	−0.10 (−0.45, 0.25)	0.572

*MD* Mean Difference, *CI * Confidence Interval, *SG * Sleeve Gastrectomy, *CPAP * Continuous Positive Airway Pressure, *AHI * Apnea–Hypopnea Index, *ODI * Oxygen Desaturation Index. Estimates represent interaction terms (Time × CPAP Duration) from generalized estimating equation (GEE) models with an exchangeable correlation structure. Each value reflects the additional change in outcome from pre- to post-surgery associated with each one-month increase in preoperative CPAP use. Negative values indicate reduced improvement with longer CPAP use; positive values indicate greater improvement. Bold italic p-values indicate statistically significant differences at *p* < 0.05

Collectively, these findings suggest that while SG confers significant benefits in airway anatomy and sleep-disordered breathing, prolonged CPAP use before surgery may reflect a more treatment-refractory phenotype with diminished capacity for postoperative reversal.

### Correlation between %TWL and Improvements in Sleep Metrics

**Fig. 6:**
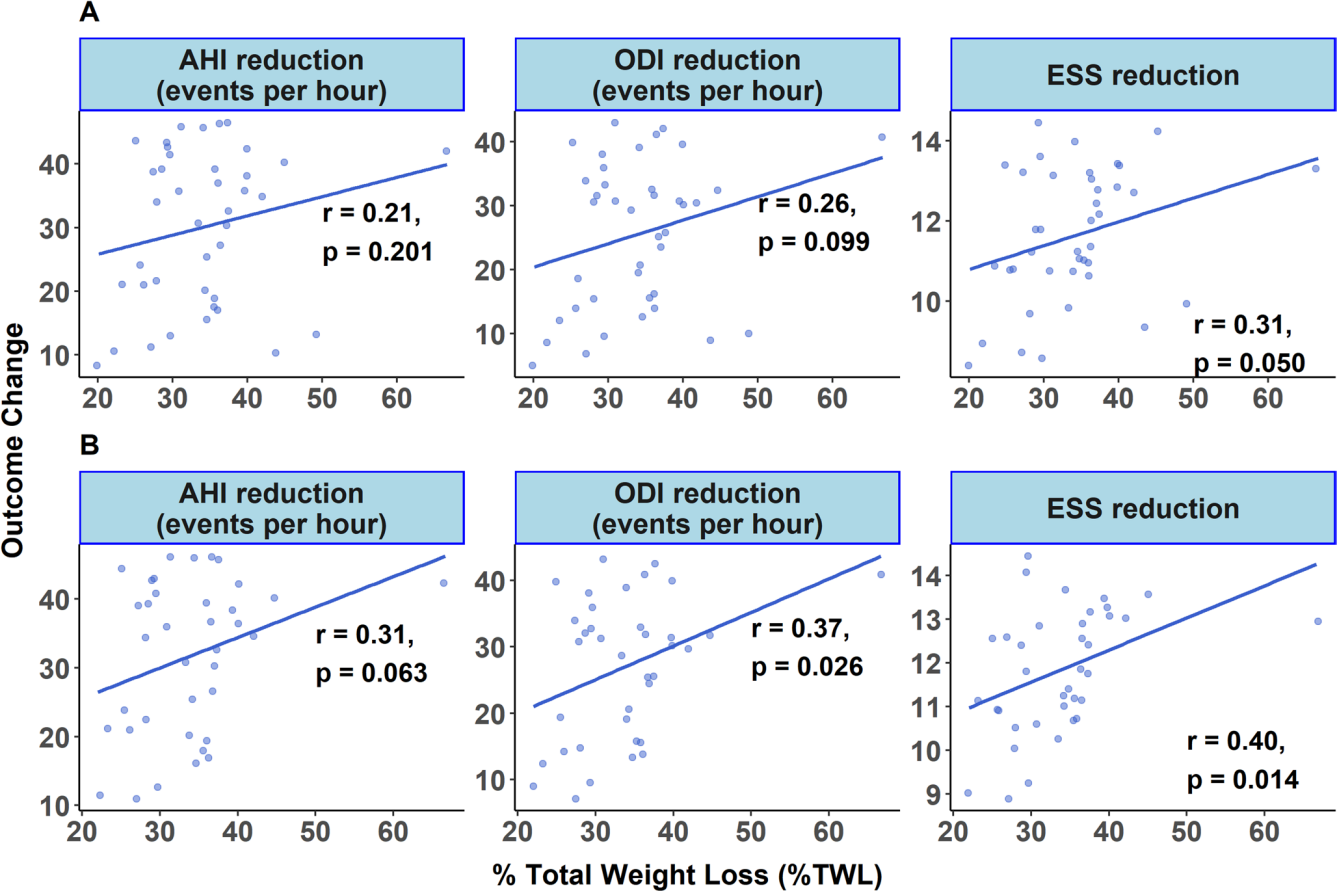
Correlation between percentage total weight loss (%TWL) and improvements in sleep-related indices across the study population. Panel A displays the correlations for the entire sample, demonstrating modest positive relationships with reductions in AHI and ODI, and a statistically significant positive correlation with the reduction in Epworth Sleepiness Scale (ESS) (*r* = 0.31, *p* = 0.050). The three data points with the highest Cook’s Distance values across all models are highlighted in red. Panel B presents the results of a sensitivity analysis excluding these same three observations. Although none were classified as highly influential (Cook’s D < 1; F-distribution percentiles < 20%), they exceeded the rule-of-thumb threshold of 4/(n – k – 1). Their exclusion resulted in strengthened associations, with improved correlations for AHI (*r* = 0.31, *p* = 0.063), ODI (*r* = 0.37, *p* = 0.026), and ESS (*r* = 0.40, *p* = 0.014). AHI – Apnea-Hypopnea Index; ODI – Oxygen Desaturation Index

In examining the relationship between %TWL and improvements in sleep-related metrics, a significant positive correlation was observed with ESS reduction (*r* = 0.31, *p* = 0.050), while correlations with reductions in AHI and ODI remained non-significant in the full sample (*r* = 0.21 and 0.26, respectively; *p* > 0.05 for both) (Fig. 6A). A sensitivity analysis was conducted after excluding the same three observations with the highest Cook’s Distance values across all models. Although these observations were not classified as highly influential (Cook’s D < 1 and F-distribution percentiles < 20%), they exceeded the commonly used rule-of-thumb threshold of 4/(N-k-1)​, justifying their consideration as potentially influential. Their exclusion revealed strengthened associations between %TWL and reductions in AHI (*r* = 0.31, *p* = 0.063), ODI (*r* = 0.37, *p* = 0.026), and ESS (*r* = 0.40, *p* = 0.014) (Fig. 6B). However, correlations between %TWL and upper airway dimensions or CPAP settings remained weak and statistically insignificant (Fig. 6).

## Discussion

MBS has emerged as an effective modality for managing obesity-related OSA [13–16, 29–31]. To our knowledge, this is among the first studies to integrate MRI-based anatomical analysis with functional polysomnographic outcomes to evaluate upper airway remodeling after SG. Studies have documented significant improvements across multiple physiological parameters in patients with OSA post-MBS. Additionally, limited studies have highlighted the anatomical alterations in the upper airway that follow MBS [17]. However, the correlation between these anatomical changes and physiological outcomes has not been fully explored. This study aimed to address this gap by evaluating the effectiveness of SG in alleviating OSA symptoms through the assessment of pre- and post-operative changes in upper airway morphology via MRI, along with analyzing sleep parameters, to correlate these objective findings with patient-reported clinical outcomes.

SG was selected as the sole intervention due to its global, national, and institutional prominence, as well as its favorable safety profile compared to hypoabsorptive procedures [32–34]. This choice ensured procedural consistency and minimized the confounding effects of anatomical and metabolic variability, allowing for a clearer evaluation of SG’s impact on upper airway remodeling.

The study population was restricted to adults to minimize physiological variability associated with age extremes. Adolescents were excluded due to ongoing neurodevelopmental changes, including cortical maturation, pubertal hormonal shifts, reductions in slow-wave activity, and circadian phase delays, all of which may influence upper airway dynamics and sleep architecture [35, 36]. Similarly, individuals over 65 years were excluded due to known age-related alterations in pharyngeal anatomy, upper airway collapsibility, reduced Rapid Eye Movement (REM) and slow-wave sleep, and declines in melatonin and testosterone levels, which may independently affect OSA severity and therapeutic response [37]. These exclusions were intended to enhance the internal validity of anatomical and polysomnographic outcomes following SG.

Our findings are consistent with prior literature demonstrating that weight loss is associated with improvements in OSA-related physiological parameters. In postoperative PSG, the average AHI decreased to 8.0 ± 0.6 events per hour, placing the majority of participants (75%) in the mild OSA range (AHI 5–14). Notably, no participants remained in the severe OSA category after treatment, and 17.5% of participants achieved a normal AHI (< 5), indicating a complete resolution of their OSA. Additionally, there were parallel improvements in the nocturnal ODI and ESS scores, which reflected reductions of 68.5% and 67.6%, respectively. Moreover, MRI findings have shown improved post-operative upper airway patency and improved airway dynamics. These findings agree with previous studies regarding the effect of bariatric surgery in reducing the severity of OSA across the participant group (28,29).

### The Impact of SG on Excessive Daytime Sleepiness

To evaluate excessive daytime sleepiness (EDS), we used the ESS, which assesses the likelihood of falling asleep in eight common situations (e.g., sitting quietly, reading, driving) [38]. Before the intervention, 75% of participants exhibited severe daytime sleepiness (ESS ≥ 16), while a smaller proportion had moderate (15%) or mild (10%) sleepiness, with no participants falling within the normal range.

This unexpectedly high burden of severe EDS (ESS ≥ 16 in 75% of participants), despite CPAP use, likely reflects the multifactorial impact of obesity on sleep quality and architecture. Obesity contributes to sleep fragmentation through mechanical upper airway narrowing, gastroesophageal reflux, systemic inflammation, and neurohormonal dysregulation [39, 40]. Moreover, chronic, untreated OSA may result in cumulative neurocognitive fatigue and sleep debt that is not readily reversed by short-term CPAP therapy [39, 41]. Notably, our post hoc analysis revealed that longer preoperative CPAP use was significantly associated with attenuated improvements in ESS, AHI, and ODI.

Following SG, ESS scores significantly improved to 5.4 ± 0.2 (*p* < 0.001), with all patients achieving ESS scores within the normal range postoperatively. This significant improvement in ESS scores is consistent with findings from previous studies, as Dilektasli et al. evaluated ESS scores for a cohort with a pre-operative ESS mean of 9.0 ± 4.6 and have shown a remarkable improvement to a mean of 3.5 ± 2.2 [42].

This 12-point reduction surpasses those reported in prior studies. For instance, Dilektasli et al. observed a 5.5-point decrease (from 9.0 to 3.5) six months after SG in a cohort with milder baseline EDS [42]. The superior outcomes in our study likely reflect more severe baseline EDS, more significant weight loss (− 34.0% TWL), and longer follow-up (14 months). Additionally, our inclusion of patients actively using CPAP, in contrast to their exclusion in the comparator study, may have contributed to a more symptomatic cohort and, thus, more pronounced improvements. Similarly, Nastałek P et al. found a 7-point reduction in ESS (from a mean of 12 to 5) in a follow-up period of 12 months [43]. However, Nastalek P et al. did not correlate their findings with the amount of weight lost [43].

The complete remission of EDS emphasizes the potential of SG as an effective adjunct intervention for individuals with obesity and OSA-related sleepiness, particularly when significant weight loss is achieved. This aligns with broader enhancements in sleep quality after SG, including fewer nocturnal awakenings, reduced snoring, and improvements in insomnia severity and fatigue [44, 45]. Wyszomirski et al. reported significant improvements in sleep-related symptoms and Athens Insomnia Scale scores post-SG [44], while Taşdöven et al. found substantial fatigue reduction and improved quality of life [45].

These findings are consistent with mechanistic studies linking weight loss to reduced upper airway resistance, improved sleep architecture, and attenuation of contributing factors such as chronic intermittent hypoxia and systemic inflammation [5, 46, 47].

### Polysomnography Outcomes and CPAP Reliance

Preoperatively, patients exhibited a mean AHI of 38.1 ± 2.5 events/hour and an ODI of 30.7 ± 2.2 events/hour, highlighting the significant burden of OSA in the MBS population, with PSG showing prevalence rates as high as 79% [44].

Post-SG, AHI improved by 78.9% to 8.0 ± 0.6 events/hour, and ODI decreased by 83.1% to 5.2 ± 0.5 events/hour, resolving all cases of severe OSA and moving 75% of patients to the mild AHI category. These outcomes corroborate findings from the meta-analysis conducted by Peromaa-Haavisto et al. [48], which also highlighted the positive impact of SG on AHI and ODI; however, our results exceeded the average improvement rates previously documented in the literature. Our observed AHI reduction surpassed the 61% average typically recorded after surgical interventions [49] and the 56.1–61.5% remission rates of OSA found in long-term SG studies [15, 16].

Our superior outcomes may stem from more rigorous patient selection, including exclusion of reflux disease, greater weight loss (− 43.8 kg; −15.7 kg/m² BMI), and the anatomical remodeling documented on MRI. Moreover, the preoperative inclusion of patients on CPAP might have bolstered severity assessments and potential improvement.

The improvements in sleep metrics reinforce how SG alleviates OSA through reduction of visceral and pharyngeal fat, decreased airway collapsibility, and metabolic enhancements. These anatomical and physiological changes likely explain why weight loss contributes to OSA remission [14, 49].

Our subgroup analysis further indicated that the magnitude of anatomical and functional improvement following SG was comparable between patients with class I–II and class III obesity. This supports prior evidence that baseline BMI alone is not a key determinant of OSA remission, and that significant improvements in airway anatomy and function can be achieved across the obesity spectrum, provided sufficient weight loss occurs [50].

In parallel, reliance on CPAP therapy dropped significantly. CPAP use decreased from 90% preoperatively to 22.5% postoperatively, and those who continued using CPAP required significantly lower pressures of − 5.3 cm H₂O, which appear greater than the reductions reported in prior studies [51, 52]. These outcomes support earlier findings by Hoyos et al., who demonstrated that substantial weight loss reduces pharyngeal collapsibility, allowing many patients to wean off CPAP entirely [53]. The decline in pressure requirements provides an objective marker of improved airway function.

Interestingly, our results revealed that longer preoperative CPAP duration was inversely associated with postoperative improvements in both functional (AHI, ODI, ESS) and anatomical (soft palate length) outcomes. Each additional month of CPAP use correlated with smaller gains, suggesting a treatment-refractory OSA phenotype. This may reflect chronic, severe OSA that induces long-standing pharyngeal tissue remodeling and neuromuscular adaptation, limiting reversibility even after substantial weight loss. Previous studies have similarly noted that greater baseline OSA severity predicts residual apnea post-MBS, implicating fixed anatomical or non-obesity-related traits such as craniofacial morphology or ventilatory control abnormalities [50].

However, variability exists across the literature. For example, Nastałek et al. reported persistent CPAP use in many patients despite physiological improvement, and van Veldhuisen et al. observed worsened CPAP compliance following surgery [43, 54]. These discrepancies may relate to differences in follow-up duration, population characteristics, and definitions of adherence. Nonetheless, our findings demonstrate that in appropriately selected patients, SG can substantially reduce both the need for and intensity of CPAP therapy, offering a meaningful improvement in quality of life.

The role of preoperative PSG in candidates for MBS remains contentious. Some researchers question its universal necessity for all patients undergoing MBS [43]. However, our data highlights the importance of PSG in the longitudinal management of OSA. Baseline PSG facilitated objective OSA diagnosis and severity stratification, while postoperative PSG validated therapeutic success. Absent this data, the degree of functional improvement could not be quantified. Furthermore, our findings advocate for the view that addressing obesity through MBS not only mitigates anatomical contributors to OSA but also addresses its metabolic drivers, resulting in high remission rates.

Moreover, our cohort, predominantly comprising females (75%) with an elevated baseline AHI, presents a notable divergence from existing literature, which typically indicates that females exhibit lower baseline AHI values compared to males [55]. Interestingly, the baseline AHI observed in our population exceeded the average reported for females in similar studies. This anomaly might be attributed to anatomical variations in the upper airway that differ across ethnic groups [56–58], potentially elucidating the increased risk of airway compressibility noted in our cohort. While the current study did not assess the ethnic backgrounds of participants, our findings underscore the necessity of considering this variable in future research endeavors.

### Anatomical Remodeling of the Upper Airway

To better understand the mechanisms driving OSA improvement after weight loss, we conducted pre- and postoperative MRI assessments of the upper airway. Baseline imaging revealed multiple anatomical risk factors associated with OSA, including a narrow posterior nasal airway space (PNAS; mean 5.9 mm), enlarged tongue volume (103 mL), and an elongated, thick soft palate (mean length 41 mm, thickness 12.5 mm). These features align with established imaging findings in OSA patients, where retropalatal and lateral pharyngeal narrowing, soft palate redundancy, and tongue base crowding are common contributors to airflow obstruction [17, 59, 60].

Dynamic MRI studies have shown that up to 80% of OSA cases involve collapse at the retropalatal level, often exacerbated by increased soft tissue mass [59, 60]. larger tongues contribute to airway crowding and are independently associated with OSA severity [17, 59]. Athayde et al. reported a correlation between greater tongue volumes and higher Mallampati scores, reinforcing the link between tongue size and airway compromise [60]. Further dynamic MRI studies have illustrated that in OSA patients, the downward movement of the tongue during sleep likely exacerbates airway obstruction [61]. Li et al. and Subasi et al. have similarly highlighted the contribution of a long, thick soft palate to retropalatal obstruction, particularly in severe OSA cases [59, 62].

Following SG, we observed substantial anatomic remodeling. Tongue volume decreased by an average of 23.4 mL, and posterior airway spaces at the nasal, occlusal, and mandibular levels increased by 3.0 mm, 3.5 mm, and 3.9 mm, respectively. The dimensions of the soft palate were also reduced (length: −4.9 mm; thickness: −3.0 mm), indicating decreased tissue redundancy in the retropalatal region. These findings reflect meaningful structural decompression of the upper airway.

Our results are in strong concordance with recent volumetric MRI studies. Sutherland et al. and Wang et al. showed that MBS leads to significant reductions in tongue and pharyngeal fat volume, thereby improving velopharyngeal airway dimensions and airflow during sleep [17, 63]. Notably, Wang et al. identified tongue fat reduction as a principal mediator of AHI improvement, independent of overall weight loss [17]. Although our imaging did not differentiate between fat and lean tissue, the magnitude of soft tissue volume reduction observed likely reflects a decrease in intra-tissue fat. This may explain why some patients experience disproportionately greater improvement in OSA severity relative to their total weight loss. Targeted reduction in pharyngeal fat yields a more favorable airway architecture and respiratory function.

The anatomical changes observed in our study mirrored the significant functional improvements seen on PSG, including reductions in AHI and ODI. These findings bridge an important gap noted in earlier studies that lacked concurrent imaging and sleep metrics. They also support the hypothesis that weight loss stabilizes airway dynamics during sleep by reducing tissue collapsibility at key obstruction sites, such as the tongue base and soft palate [17, 63]. Thus, MRI analysis demonstrates that SG produces favorable enhancements in upper airway anatomy, which may contribute to improved respiratory function.

### Weight Loss and Associated Medical Conditions

The impact of %TWL extended beyond respiratory metrics, showing strong correlations with improvements in the ESS, AHI, and ODI, with correlation coefficients of 0.37 to 0.47 after excluding outliers. This indicates a dose-response relationship: greater weight loss leads to more significant improvements in sleep-disordered breathing and daytime alertness. Even moderate weight loss eliminated severe OSA in all cases, with 75% of patients achieving a mild AHI postoperatively. These findings underline the importance of maximizing weight loss through postoperative support to enhance OSA outcomes.

Despite anatomical improvements seen on MRI, these changes did not predict individual sleep function outcomes, indicating that static anatomical remodeling isn’t the only factor in OSA remission. Instead, mechanisms like reductions in visceral fat, improved lung volumes, and reduced systemic inflammation are likely significant contributors. This dissociation between structural and functional outcomes is consistent with prior OSA research, which has shown that up to 35% of patients may have residual OSA after MBS, despite significant weight loss, emphasizing that obesity is a major but not exclusive contributor to OSA pathogenesis [64]. A recent meta-analysis by Oweidat et al. reported significant reductions in BMI and AHI after MBS, with a 65% OSA remission rate, highlighting the role of weight loss while also suggesting the involvement of additional non-obesity-related etiologies in residual OSA [64].

SG also led to significant improvements in obesity-related diseases: complete remission in all patients with type 2 diabetes or hypertension, improvement in dyslipidemia in 85.7% of cases, and positive outcomes in cardiac conditions. These results align with previous reports showing high remission rates for associated diseases post-MBS, attributed to improved insulin sensitivity and reduced hepatic lipogenesis [20, 65, 66].

Osteoarthritis was prevalent in the cohort, with symptom improvements but limited full remission due to structural joint changes. OA can disrupt sleep and exacerbate OSA symptoms [46, 67], and conditions like dyslipidemia and hypertension have been linked to worsening OSA severity [5, 6].

### Strengths and Limitations

This study offers valuable insights into managing obesity and OSA. It emphasizes addressing the root causes of obesity rather than relying solely on CPAP therapy. Significant weight loss is shown to improve OSA treatment, but weak correlations between weight loss and changes in airway dimensions suggest the benefits may not be purely anatomical. Comprehensive post-surgical support is crucial for sustained weight loss.

The study’s strengths lie in its multidimensional approach, integrating subjective measures (ESS), objective diagnostics (PSG and nocturnal oximetry), and structural imaging (MRI), which enhance understanding of weight loss effects on airway patency. Detailed MRI metrics facilitate anatomical quantification of airway changes, linking tissue volume reduction to functional improvements. Additionally, practical clinical parameters like CPAP pressure and compliance rates make the findings applicable to patient-centered care. Correlation analysis shows a dose-response relationship between %TWL and sleep metric improvements (AHI, ODI, ESS), indicating these trends are consistent across most patients.

However, this study has several limitations. The follow-up duration of approximately 14 months, while valuable, may be insufficient to assess the long-term durability of OSA improvement, especially considering the potential for recurrence with weight regain, aging, or evolving comorbidities [50]. Additionally, the study refrained from comparing outcomes across various MBS procedures. While SG is recognized as an effective and the most commonly performed MBS procedure [33], the comparative efficacy of gastric bypass remains a pertinent question, especially as some data indicate differing remission rates for OSA [68, 69]. Moreover, the study lacked a control group, such as patients undergoing non-surgical weight loss or alternative interventions. As a result, we cannot definitively attribute improvements to SG alone; the findings demonstrate association rather than causation. The absence of blinding may also introduce expectancy effects, particularly for subjective outcomes like ESS.

While methodologically sound, our sample size was modest and predominantly female (75%), which could restrict the generalizability of the findings, given the known sex-based differences in OSA pathophysiology [55]. The study focused on respiratory and anatomical outcomes but did not assess broader OSA-related domains such as quality of life, neurocognitive function, or pharyngeal neuromuscular dynamics. MRI was conducted during wakefulness, not sleep, and did not include dynamic assessments; therefore, airway enlargement should be interpreted as a surrogate rather than a direct measure of functional improvement. Factors like head position and respiratory phase during MRI were also not standardized.

Lastly, the correlation between weight loss and improvements in sleep metrics became significant only after excluding outliers, suggesting some variability in treatment response. Variables such as craniofacial anatomy, tongue fat, neuromuscular tone, and ethnicity—which may influence OSA remission—were not evaluated and warrant further investigation. This limitation is particularly evident as the study did not address variations in ethnic backgrounds that may exhibit differing anatomical characteristics of the upper airway. Future clinical research should explore these variables to elucidate the complexities of OSA remission in the context of MBS [56–58].

### Future Clinical and Research Implications

The findings of this study have substantial implications for clinical applications and future research initiatives. Clinically, the findings confirm that SG can produce significant short-term enhancements in OSA severity, overall sleep quality, and dependence on CPAP, accompanied by systemic advantages such as improved metabolic health. These results advocate for considering MBS as a primary therapeutic intervention for patients struggling with moderate-to-severe OSA and obesity, particularly in scenarios where conventional therapies are ineffective or poorly tolerated.

From a research perspective, this study highlights the importance of a multidisciplinary and mechanistic framework when examining interventions for OSA. Future research should focus on stratifying patients based on the specific type of MBS procedure to ascertain whether different surgical methodologies yield distinct rates of OSA remission. Longitudinal studies with extended follow-ups are essential to evaluate the sustainability of these improvements and the potential for OSA recurrence over time. Investigations utilizing more diverse populations will be crucial for identifying reliable predictors of surgical efficacy, including variables such as age, sex, ethnicity, craniofacial anatomy, and fat distribution within the upper airway.

The utilization of MRI to assess anatomical changes is a strength, but it may carry some limitations. The MRI was performed while awake with static images. This provides measurements of airway caliber and tongue volume, yet does not capture dynamic airway collapse during sleep. Incorporating diverse imaging techniques, including dynamic MRI, cone beam CT, and functional endoscopy, into future research protocols will aid in elucidating the anatomical and physiological mechanisms that contribute to airway improvements. Additionally, exploring metrics such as tongue fat content and assessing neuromuscular responsiveness during sleep could reveal new therapeutic avenues that extend beyond weight loss alone.

## Conclusion

This prospective study suggests that SG can produce meaningful short-term improvements in OSA severity and related comorbidities in patients with obesity. These benefits appear to be driven by both substantial weight loss and anatomical remodeling of the upper airway. Interestingly, the extent of weight loss was not significantly correlated with individual improvements in sleep function, indicating the multifactorial nature of OSA response. SG was also associated with complete resolution of daytime sleepiness and reduced CPAP reliance, in addition to metabolic improvements such as remission of diabetes and hypertension.

While these findings are promising, they must be interpreted with caution due to the limited sample size. Future research with larger cohorts and longer follow-up periods is essential to validate the observed effects and to guide patient selection and management strategies.

## Supplementary Information

Below is the link to the electronic supplementary material.

Supplementary Material 1 (DOCX 18.9 KB)

## Data Availability

No datasets were generated or analysed during the current study. The datasets generated and/or analyzed during the current study are available from the corresponding author upon request.

## Abbreviations

AHI: Apnea–Hypopnea Index
ArESS: Arabic version of the Epworth Sleepiness Scale
BMI: Body Mass Index
CPAP: Continuous Positive Airway Pressure
EDS: Excessive Daytime Sleepiness
EGD: Esophagogastroduodenoscopy
ERS: European Respiratory Society
ESS: Epworth Sleepiness Scale
GEE: Generalized Estimating Equations
IRB: Institutional Review Board
MBS: Metabolic and Bariatric Surgery
MLSP: Maximum Length of the Soft Palate
MPAS: Mandibular Posterior Airway Space
MRI: Magnetic Resonance Imaging
MTSP: Maximum Thickness of the Soft Palate
ODI: Oxygen Desaturation Index
OPAS: Occlusal Posterior Airway Space
OSA: Obstructive Sleep Apnea
PNAS: Posterior Nasal Airway Space
PSG: Polysomnography
RYGB: Roux–en–Y Gastric Bypass
SE: Standard Error
SG: Sleeve Gastrectomy
SDB: Sleep–Disordered Breathing
TWL: Total Weight Loss
