# Supplementary material for: Anatomical Remodeling of the Upper Airway after Laparoscopic Sleeve Gastrectomy: A Multimodal Assessment of Structural and Functional Improvements in Obstructive Sleep Apnea
Source: Obes Surg. 2025 Nov 13;35(12):5316–35. doi: 10.1007/s11695-025-08352-z (PMC12722346; doi:10.1007/s11695-025-08352-z)
Supplement: Supplementary file 1 — Supplementary Material 1 (DOCX 18.9 KB) [file 11695_2025_8352_MOESM1_ESM.docx]

# Supplementary Data

| **Supplementary Table S1 : Lab investigations pre and post-SG.** | | | | |
| --- | --- | --- | --- | --- |
| **Variable** | **Pre-operative**  **(M±SE)** | **Post-operative**  **(M±SE)** | **MD (95% CI)**  **(After-Before)** | ***p*** |
| **Lab investigations** |  |  |  |  |
| Hemoglobin (g/dL) | 12.6 ± 0.2 | 12.3 ± 0.2 | -0.3 (-0.9, 0.3) | 0.371 |
| Ferritin (ng/mL) | 121.8 ± 16.3 | 107.8 ± 28.4 | -14.0 (-77.4, 49.4) | 0.665 |
| WBC (×10³/μL) | 8.2 ± 0.5 | 6.6 ± 0.4 | -1.6 (-2.8, -0.4) | 0.007* |
| PLT (×10³/μL) | 292.4 ± 10.2 | 284.1 ± 10.5 | -8.4 (-36.7, 20.0) | 0.563 |
| INR | 1.0 ± 0.0 | 1.0 ± 0.0 | 0.0 (0.0, 0.0) | 0.087 |
| AST (U/L) | 26.1 ± 1.1 | 19.0 ± 1.3 | -7.1 (-10.5, -3.7) | < 0.001* |
| ALT (U/L) | 27.6 ± 1.3 | 21.4 ± 2.3 | -6.2 (-11.3, -1.1) | 0.017* |
| CRT (mg/dL) | 0.9 ± 0.0 | 0.8 ± 0.0 | -0.1 (-0.2, 0.0) | 0.027* |
| Uric acid (mg/dL) | 4.5 ± 0.2 | 4.4 ± 0.2 | -0.1 (-0.6, 0.4) | 0.738 |
| Urea (mg/dL) | 28.6 ± 1.6 | 30.5 ± 1.1 | 1.9 (-1.9, 5.8) | 0.317 |
| fT3 (pg/mL) | 3.6 ± 0.4 | 3.2 ± 0.1 | -0.3 (-1.0, 0.4) | 0.396 |
| fT4 (ng/dL) | 2.3 ± 0.6 | 1.3 ± 0.0 | -1.0 (-2.2, 0.2) | 0.097 |
| TSH (μIU/mL) | 2.7 ± 0.3 | 2.7 ± 0.2 | 0.0 (-0.7, 0.7) | 0.996 |
| Fasting glucose (mg/dL) | 98.5 ± 3.8 | 83.1 ± 1.5 | -15.4 (-23.4, -7.5) | < 0.001* |
| HbA1c (%) | 5.7 ± 0.1 | 5.1 ± 0.1 | -0.6 (-0.9, -0.3) | < 0.001* |
| Fasting Insulin (μIU/mL) | 19.9 ± 0.5 | 17.6 ± 0.7 | -2.3 (-4.0, -0.6) | 0.008* |
| HOMA IR | 1.0 ± 0.0 | 1.0 ± 0.1 | 0.0 (-0.2, 0.2) | 0.924 |
| TG (mg/dL) | 147.6 ± 12.3 | 103.7 ± 3.5 | -44.0 (-68.7, -19.3) | < 0.001* |
| Cholesterol (mg/dL) | 183.9 ± 5.3 | 157.1 ± 3.8 | -26.8 (-39.4, -14.3) | < 0.001* |
| LDL (mg/dL) | 82.6 ± 2.3 | 65.2 ± 3.3 | -17.4 (-25.2, -9.6) | < 0.001* |
| Albumin (g/dL) | 4.5 ± 0.1 | 4.1 ± 0.1 | -0.4 (-0.6, -0.1) | 0.003* |
| Calcium (mg/dL) | 9.3 ± 0.1 | 9.2 ± 0.1 | -0.1 (-0.4, 0.2) | 0.564 |
| Vit D (ng/mL) | 42.8 ± 1.3 | 30.6 ± 1.9 | -12.2 (-16.6, -7.7) | < 0.001* |
| VIT B12 (pg/mL) | 602.4 ± 33.9 | 697.4 ± 201.8 | 95.1 (-301.0, 491.1) | 0.638 |
| PTH (pg/mL) | 39.4 ± 2.4 | 40.6 ± 2.4 | 1.2 (-5.3, 7.7) | 0.719 |

Cell values before and after represent mean ± standard error (M±SE). MD: mean difference between post and pre-surgical values. CI: confidence intervals. *Statistically significant (p < 0.05). **BMI**: Body Mass Index; **TWL**: Total Weight Loss; **CPAP**: Continuous Positive Airway Pressure; **HbA1c**: Glycated Hemoglobin; **WBC**: White Blood Cells; **PLT**: Platelet Count; **INR**: International Normalized Ratio; **AST**: Aspartate Aminotransferase;**ALT**: Alanine Aminotransferase; **CRT**: Creatinine; **TG**: Triglycerides; **LDL**: Low-Density Lipoprotein; **Vit D**: Vitamin D; **Vit B12**: Vitamin B12; **PTH**: Parathyroid Hormone; **TSH**: Thyroid Stimulating Hormone.
